# Adoption of electronic medical records in developing countries—A multi-state study of the Nigerian healthcare system

**DOI:** 10.3389/fdgth.2022.1017231

**Published:** 2022-11-21

**Authors:** Christie Divine Akwaowo, Humphrey Muki Sabi, Nnette Ekpenyong, Chimaobi M. Isiguzo, Nene Francis Andem, Omosivie Maduka, Emem Dan, Edidiong Umoh, Victory Ekpin, Faith-Michael Uzoka

**Affiliations:** ^1^ Community Medicine Department, University of Uyo, Uyo, Nigeria; ^2^ Health Systems Research Hub, University of Uyo Teaching Hospital, Uyo, Nigeria; ^3^ ICT Department, The ICT University, Yaounde, Cameroon; ^4^ Community Health Department, University of Calabar, Calabar, Nigeria; ^5^ Department of Surgery, Federal Medical Centre, Owerri, Nigeria; ^6^ Hopsital’s Management Board, Akwa Ibom State Ministry of Health, Uyo, Nigeria; ^7^ Department of Preventive and Social Medicine, University of Port Harcourt Teaching Hospital, Port Harcourt, Nigeria; ^8^ Fisheries and Aquaculture, University of Uyo, Uyo, Nigeria; ^9^ Department of Mathematics and Computing, Mount Royal University, Calgary, Alberta, Canada

**Keywords:** electronic medical records, digital health, medical informatics, adoption of eHealth, Nigeria, developing countries

## Abstract

Electronic medical records (EMR) are extensively used in developed countries to manage patient records and facilitate consultations and follow-up of treatment. This has resulted in centralised databases where different services and clinicians can quickly access patient data to support healthcare delivery. However, adoption and usage of EMR in developing countries is not common and, in most cases, non-existent. Clinicians are dependent on patients keeping their own records manually with no centralised database to manage and control the patient medical history. The key objective of this study was to investigate the propensity of clinicians and senior management personnel in healthcare facilities to adopt EMR and evaluate the contextual factors that impact or impede adoption. Using Davis's technology adoption model extended with other factors, this study determined if contextual or situational factors are associated with barriers that impede adoption of EMRs in developing countries. Using a cross-sectional quantitative research approach, a questionnaire was designed to collect data across four states in the Niger Delta region of Nigeria. Stratified random sampling was used to select healthcare facilities that participated in the survey and selection of respondents from each healthcare facility. Data was collected by trained research assistants and a total of 1,177 valid responses were received and analysed using factor analysis and multiple regression analysis. The results from the analysis show that usefulness, critical success factors, awareness and relative advantage significantly influence clinicians' intention to adopt EMRs. Surprisingly, infrastructure availability was not statistically significant. Meanwhile, risk and data security both negatively influence adoption, indicating that user perception of risk and safety of their data decreases their propensity to adopt EMRs. The results from this study suggests that usefulness and anticipated success factors in facilitating operations within healthcare facilities have a great influence on user adoption of EMRs. Awareness, training and education of users on the effectiveness of EMRs and their usefulness will increase adoption. The results will be beneficial in helping government and healthcare leaders formulate policies that will guide and support adoption of EMR. Other policy recommendations and suggestions for future research were also proffered.

## Introduction

The health sector has recently seen numerous technological innovations designed to streamline the process of healthcare delivery for the health providers and consumers. Electronic medical record (EMR) is one of such innovations. This innovation has helped to reduce medication errors, adverse drug reactions, and to improve compliance to practice guidelines among health care professionals (1). For instance, by making patients’ information available electronically, health care information systems can help to prevent ordering of duplicate tests and procedures, thereby reducing expenditures on health care service (2). Hence, these innovations have been crucial to improving patient safety and quality of health in today's world.

The usual entry point for information technology (IT) in the health sector is the EMR. EMRs are increasingly being utilized worldwide, especially in developed countries. Developing countries have been found to lag behind due to organizational, financial and infrastructural factors (3). The national e-health strategy toolkit which was developed by WHO and ITU has defined EMR as *a computerized medical record used to capture, store, and share information among healthcare providers in an organization, supporting the delivery of health services to patients* (4)*.* EMRs have been proven to improve quality of care and patient outcome by keeping health care providers better informed, improving the workflow process, improving communication between clinicians, improving compliance with best practices and reducing medical errors. Moreover, EMRs also allow patients to access their own medical records easily and from research stand point, it allows easy access to data (5–7).

In both public and private sectors, health care providers are being encouraged to migrate from paper-based health records to electronic storage of patient information and computer-aided decision support systems. This is partially due to a growing recognition that a better information technology infrastructure is essential to addressing certain health-related national concerns such as the need to improve the safety and quality of health care, and rising health care costs (5). However, adoption of EMR among health workers has been slow in developing countries like Nigeria. Adoption is of course more than designing or purchasing a reasonably functional technology, but also about the acceptance and use of the system by the health workers.

Recent reports show that the fit between information technology and the clinical work system will lead intended end users to accept or reject it, to use it or misuse it, to incorporate it into their routine or work around it (8). The successful implementation of innovative medical technologies, depends on acceptance by medical staff, such as doctors, nurses and other clinicians. The decision to accept or reject a new technological innovation depends on several influencing factors. For example, Ross et al. in a review of forty-four studies from North America and Europe identified barriers to implementation of EMR as adaptability, complexity, cost, external policy and incentives (9). Other contextual factors include organizational and social environments, cultures, state of the economy, medical liability and processes by which innovation is introduced (10, 11). Other barriers identified are personal attributes such as knowledge, beliefs, computer skills, experience, resistance to change and lack of health care providers' input in design and implementation of the EMR (12, 13). An effective EMR is one that serves its intended purposes after implementation, hence uptake of an EMR is just as important its performance.

Several studies have analysed the behavioural intentions of healthcare professionals to accept and use a new health information technology. In van der Meijden et al. (14), several factors which influenced the success of health information systems were reported, including: System quality attributes like ease of use, response time, useability; information quality attributes like completeness, accuracy of data, legibility; Usage attributes like number of entries, frequency of use, duration of use; User attributes like user satisfaction, attitude, user friendliness; Individual impact attributes like changes to work patterns, documentation frequency, time of day for documenting; and organizational impact attributes such as impact on patient care, communication and collaboration, reduction of staff as well as time saving (14). Chae et al. (15) reported that larger hospital size and having top management support was significantly associated with adoption and utilization of EMR by health professionals. This may be because as hospital sizes get bigger, the adoption rate of EMR increased to improve efficiency of hospital operations and patient management (15).

A mismatch between EMR functionality and the needs of health workers in practice was identified as a common deterrent to its uptake in O’Donnell et al. (13). EMRs were often viewed as lacking an easily accessible overview of key patient data such as family histories and past medical histories. Furthermore, it was reported that physicians found it difficult to record certain types of information such as emerging diagnoses and/or vague symptoms, especially for potentially sensitive or stigmatising diagnoses (13). In Nigeria, although positive attitudes and perception towards EMR was seen in the Northwest, poor knowledge was prevalent among the health workers, especially among the doctors (16). This therefore can be a major barrier to the adoption and utilization of EMR by health professionals. A review of the adoption of electronic health records (EHR) in Africa also found that it has not been widely implemented or adopted in sub-Saharan Africa (17). In another study carried out in Cameroon to develop and test an EHR system locally, it was found that there are many contextual challenges which required modelling of the system to fit the local medical practice in place and using terminology that is tailored and appropriate for their operations (18).

The main objective of this study is to investigate the factors that influence the decision of healthcare professionals to adopt and use an EMR system by answering the following research question: *What are the factors that influence the decision of health workers to adopt and use EMR in developing countries?*

Different models have been theorized to examine adoption of technology in an organizational setting. These have been done with the intent of understanding the attitudes, the use intentions and the behaviour towards the adoption of new technologies (11). One of the most notable is the application of the Technology Acceptance Model (TAM) to the prediction and explanation of end-user reactions to health IT. TAM is based on the theory of reasoned action (TRA) and hypothesizes that Perceived Usefulness and Perceived Ease of Use, are of primary relevance for technology acceptance. How useful technology is perceived to be is subjective and depends on how the individual thinks their jobs will be enhanced by the technology, while perceived ease of use is the subjective assessment of the effort required to learn and use the new technology. The balance between these two determines the acceptance of the technology by individuals, and their motivation to use the technology. External factors, such as support measures, have a positive effect on the perception of usefulness and on understanding a technology (11, 16). In a review of TAM usage in health care, it was able to predict 30%–70% of variance of behavioural Intention to Use (16).

Another of such theories is the Theory of Reasoned Action which was developed by Martin Fishbein and Icek Ajzen in 1975. It is a popular model from social psychology which is concerned with the determinants of consciously intended behaviours (19). TRA was proposed after the researchers had difficulty explaining the discrepancy between attitude and behaviour in previous models. The model was developed to explain whether individual behaviour such as adoption and utilization of a new technology is driven by behavioural intentions, with individual attitude, subjective norms surrounding the performance of the behaviour and ease of use of the technology affecting behaviour (19). Theory of planned behaviour has also been employed in explaining models of adoption of technology and it goes a step further than the TRA by incorporating “perceived behavioural control”, accounting for situations where the target individuals don't have total control over the behaviour (20).

The unified technology acceptance and use of technology (UTAUT) model was published in 2003 and is based on an analysis and comparison of up to eight technology acceptance models, including TAM, TAM2, the Theory of Reasoned Action and the Diffusion of Innovation Theory (21). This theory describes 4 major factors that underpin acceptance of health technology: performance expectancy, effort expectancy, social influence, and facilitating conditions. Performance expectancy is the expectation that the system will do the job it was set out to do, and bring about improvement. Effort expectancy is the expectation that the system is easy to use. Social influence is the perception of the extent to which others believe a new technology should be used and lastly, facilitating conditions are defined as the degree to which a user believes that an organizational and technical infrastructure can support the use of the new technology (11, 19, 20).

**Figure 1:**
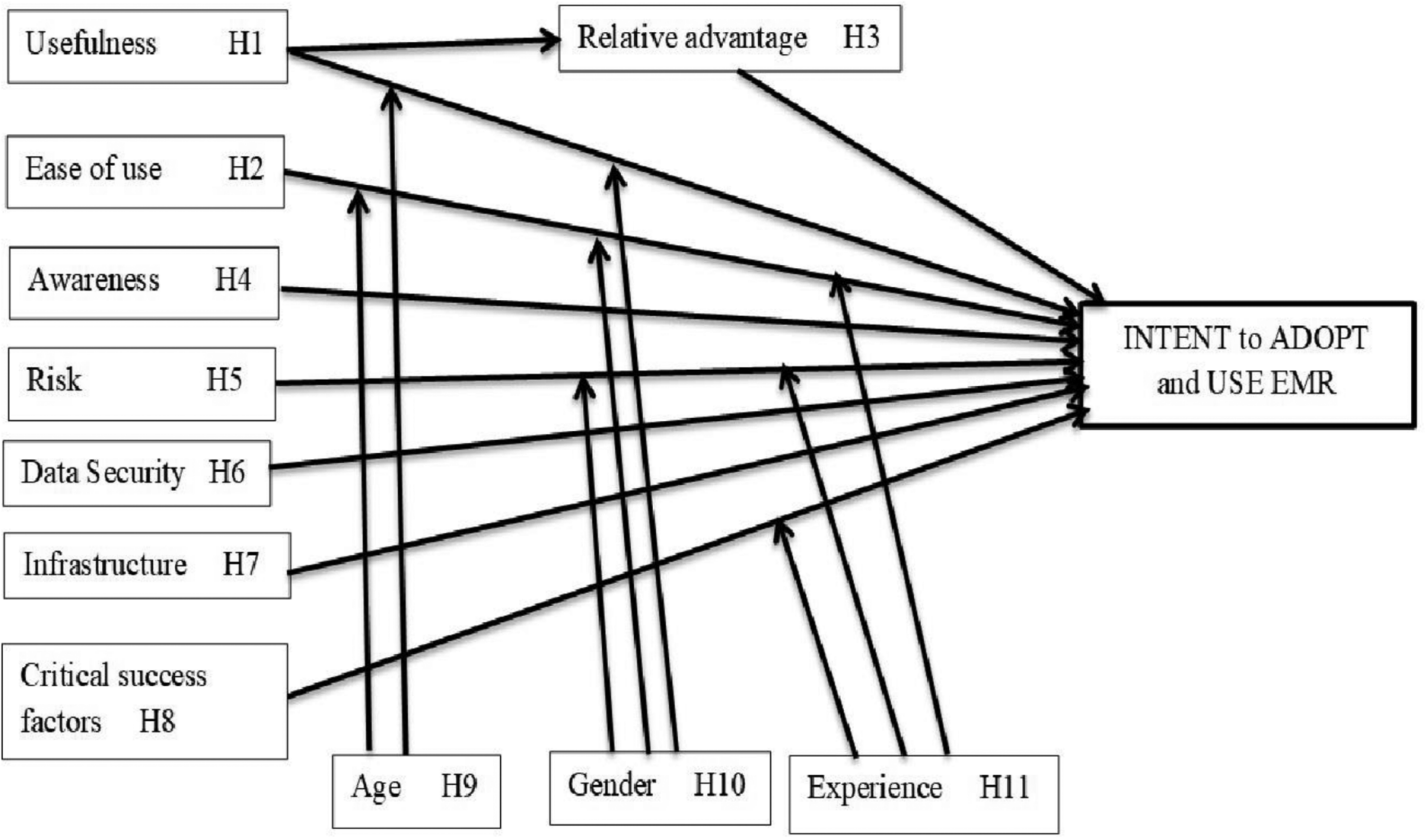
EMR adoption model.

The technology acceptance model (TAM) (22) has been extended with additional contextual and situational constructs for this study. TAM posits that adoption of new technology is influenced by user's perception of the usefulness of the new technology and ease of use of the new technology. The ability to explain individual behavioural intentions to use new technology is based on its usefulness and ease of use, and has been widely used by many information systems (IS) researchers to explain adoption of many technologies (23–25). We have extended the TAM constructs with awareness, risks, data security, infrastructure and relative advantage to investigate the propensity of healthcare workers in medical facilities in Nigeria to adopt and use EMRs. A proposed conceptual adoption model has been developed based on the chosen theoretical model (TAM) and the additional factors used to extend the model for our research as shown in Figure 1. The model shows the relationship between the independent variables that can influence adoption of EMR and user decision to adopt and use EMR. The model proposes that relative advantage mediates usefulness of EMR and hence impacts user propensity to adopt and use EMR. The model also shows the moderating factors that can influence user decision to adopt EMR such as age, gender and years of experience in the medical field.

We proposed the following hypotheses which will be tested in the data analysis to answer our key research question on the critical factors that influence the adoption and usage of EMR in developing countries. The hypotheses address the relationships between the independent variables that impact adoption of EMR and the dependent variables “intent to adopt EMR” and “intent to use EMR”.

The main reason individuals, organizations and governments embark on adoption of new technology is to facilitate their work and improve performance and profitability. Adoption of new technologies often requires a change in the status-quo and doing things differently. The ease of use and usefulness of new technology greatly determines user willingness to adopt and use the technology (22). The way new technology will be more efficient than previous methods and help users gain advantage over its competitors hence it will influence the decision to adopt the technology. We therefore posit that:

H1: Usefulness will positively influence user decision to adopt EMR
H2: Ease of use will positively influence user decision to adopt EMR
H3: Relative advantage will positively influence user decision to adopt EMR

Users will usually adopt and use technology that they have witnessed being used by others or have access to observe the new technology in action. Since some of these new technologies can impact user operations if not used correctly, users will usually be sceptical to adopt and use it without having prior knowledge and visibility of the benefits of using the new technology (26). Hence, we posit that:

H4: Awareness will positively influence user decision to adopt EMR

Users of new technology are usually apprehensive about the security risks and safety of their information as it is stored and transmitted through the internet or cloud (27). Despite the growing popularity of EMR due to its ability to allow quick and easy access to patient data, users are still reluctant to adopt it due to data security and privacy concerns (28). We posit that:

H5: Absence of risk will positively influence user decision to adopt EMR
H6: Data safety will positively influence user decision to adopt EMR

Infrastructural availability, which encompasses stable electricity supply, good telecommunication networks, and high-speed internet or broadband networks, play a crucial role in the smooth operation of EMR. Sub-Sahara African countries have very poor infrastructures and have been faced with problems of stable electricity over the years. Availability of appropriate infrastructure is crucial for the adoption of new technological innovations (29). We posit that:

H7: Availability of good infrastructure will positively influence user decision to adopt EMR

In order for senior management and other stakeholders to recommend or adopt new technology, there must be some critical success factors that are used to guide their decision. Technologies that align favourably with all or most of the critical success factors outlined by the organisation will most likely be adopted. Hence, we posit that:

H8: Alignment of EMR technology with business defined critical success factors will positively influence user decision to adopt EMR

Previous studies have highlighted key innovation diffusion factors which include relative advantage and emphasised the impact of relative advantage on user perception of usefulness of new technology (30). Therefore, we posit that:

H9: Relative advantage positively mediates user decision to adopt and use EMR

Research has shown that age, gender and years of experience of users can have an impact on their decision to adopt and use new technology (31). Moreover, users that are already comfortable with a particular system or way of working for years might be reluctant to change and adopt a new system (30). Meanwhile, it is very common for younger people and male users to adapt and adopt new technology. We therefore posit that:

H10a: Age will moderate impact of usefulness on user decision to adopt EMR
H10b: Age will moderate impact of ease of use on user decision to adopt EMR
H10c: Gender will moderate impact of usefulness on user decision to adopt EMR
H10d: Gender will moderate impact of ease of use on user decision to adopt EMR
H10e: Gender will moderate user perception of risk on their decision to adopt EMR
H10f: Experience will moderate impact of ease of use on user decision to adopt EMR
H10g: Experience will moderate user perception of risk on decision to adopt EMR
H10h: Experience will moderate user perception of success factors on decision to adopt EMR

## Materials and methods

We adopted a quantitative research approach for this study because it allowed us to efficiently collect responses from a large sample across four states in Nigeria in a cost-effective and timely manner (32). A survey instrument was designed and used for the data collection. Prior to using the survey tool for the data collection, it was peer reviewed by adoption experts and a pilot test carried out to ensure the reliability and validity of the tool for our data collection process. Research assistants were recruited from each state and trained on the data collection tool. The questionnaire was transformed and then loaded unto the ODK (Open data kit) which was used by the research assistants for data collection. The ODK is an easy-to-use data collection app that runs on the android operating system. The advantage of using the ODK is that data can be collected and stored when the tablet is offline and when connected to the internet, the data is automatically saved on a central database (33). Each research assistant was given an android tablet with the ODK running on it for the data collection.

This study was conducted in four states in the Niger Delta region viz: Akwa-Ibom, Cross Rivers, Rivers and Imo states. A Stratified random sampling method was used to select respondents for this study. The target population was all medical facilities in the four states purposefully selected for the study based on accessibility and budget availability. Using a two-step stratification process, the researchers came up with a stakeholder grouping to ensure all stakeholders were represented in the data collection. End users of EMR being Clinicians (doctors, nurses, laboratory scientists/technologists and Pharmacists) and administrators at managerial level were selected from the secondary and tertiary medical facilities. At the primary health facilities, the officers in charge and the community health extension workers (CHEWs) were the respondents.

Respondents were randomly selected from the various stakeholder groups in each medical facility. The survey was administered by the research assistants and responses entered into the ODK. Self-administration was not used because respondents were not trained on how to use the ODK. The data collection was carried out over a period of 6 weeks and all data was then collated in the ODK database and transferred into an excel spreadsheet for processing and analysis. In total, 1,177 valid responses were received for analysis.

### Data analysis

Statistical Package for Social Sciences (SPSS) was used to perform the data analysis. A reliability test on the full dataset resulted in a Cronbach Alpha value of 0.94, hence confirming the reliability of the data for further analysis. The data analysis and results were divided into three sections to cover descriptive analysis, factor analysis and multiple regression analysis.

### Descriptive analysis

Respondent profiles and the various characteristics of their medical facilities are shown in Table 1. Most of the respondents were female (60.7%) with the majority falling in the 31–50 years old age group (69%) which is consistent with the optimal working age group in the country. Most of the medical facilities had public ownership (58.4%). It was interesting to see that most of the medical facilities (46.6%) depend on the administration to make decisions on new technology adoption while very few (6.7%) relied on their IT department for such decisions. The analysis also shows that more nurses (37%) responded to the survey followed by doctors (28%) and 72% did not provide any rank. Only medical doctors are given Ranks, hence only the 329 medical doctors provided responses for rank while others left it blank.

**Table 1:** Descriptive statistics (*N* = 1,177).

	Frequency	Percentage		Frequency	Percentage
**Gender**			**Age Group**		
Male	462	39.3	21–30	139	11.8
Female	715	60.7	31–40	426	36.2
			41–50	386	32.8
**State**			51–60	218	18.5
AkwaIbom	295	25.1	>60	8	0.7
Cross River	254	21.6	**Experience**		
Imo	285	24.2	<1	17	1.4
Rivers	343	29.1	1–5	233	19.8
Others			6–10	348	29.6
			11–15	304	25.8
**Job Title**			16–20	86	7.3
Admin	83	7.1	>20	189	16.1
Doctor	329	28			
Nurse	435	37	**Qualification**		
Pharmacist	119	10.1	Diploma	143	12.1
Lab Tech	165	14	BSc	552	46.9
Other	46	3.9	MSc	216	18.4
			PhD	41	3.5
**Computer Level**			Fellowship	112	9.5
Beginner	208	17.7	Other	113	9.6
Intermediate	641	54.5			
Advance	286	24.3	**Rank**		
Expert	42	3.6	Officer	13	1.1
			Registrar	36	3.1
**Ownership**			S Registrar	46	3.9
Public	687	58.4	Consultant	57	4.8
Private	398	33.8	S Consultant	14	1.2
Mission	92	7.8	Ch Consultant	14	1.2
			Med Officer	122	10.4
**Classification**			S Med Officer	21	1.8
Primary	287	24.4	Principal Med Off	6	0.5
Secondary	487	41.4	No Rank	848	72
Tertiary	403	34.2			
			**Patient Numbers**		
**Facility Age**			<20	358	30.4
<5	36	3.1	21–50	356	30.2
5–10	244	20.7	51–100	236	20.1
11–20	317	26.9	101–200	133	11.3
21–50	435	37	201–300	52	4.4
>50	145	12.3	301–400	21	1.8
			401–500	12	1
**No of Facilities**			>500	9	0.8
None	12	1			
One	32	2.7	**Tech Selection**		
Two	59	5	Admin	549	46.6
Three	127	10.8	IT Dept	79	6.7
>Four	736	62.5	Committee	254	21.6
Unknown	211	17.9	Government	237	20.1
			Other	58	4.9

### Factor analysis

In order to perform factor analysis on our data, we executed the Kaiser-Meyer-Olkin (KMO) test of sampling adequacy and Bartlett's test of sphericity. This resulted in a KMO value of 0.940 and a Bartlett's test of sphericity (approx. Chi-square) value of 24,369.354 (Appendix B). The KMO value is above the 0.6 threshold required for good factor analysis and lies in the “marvellous” range (>0.9) as defined by Kaiser and Rice (34). Moreover, the result was significant (*p* < .001) indicating that our variables are significantly correlated and the sampling was adequate to perform factor analysis.

Exploratory factor analysis was performed on the dataset using principal component analysis (PCA) in order to extract the significant variables that impact user propensity to adopt EMR. The analysis was performed on 31 measured variables from our survey tool resulting in five factors being extracted (see Appendix C), which had eigenvalues greater than 1 (35). This is further supported by the scree plot (Appendix D) which shows the five factors retained with eigenvalues ≥1 based on the eigenvalue cut-off rule. However, it is worth noting that all the proposed components for this study loaded under the 5 factors retained after the factor analysis. Hence, regression analysis was carried out on the full dataset since no components were eliminated by the factor analysis.

The correlation matrix shown in Appendix E was used to determine if there was correlation between the component's variables or not. According to Tabachnick and Fidell (36), if the absolute value of correlations exceeds.32, then there is a 10% (or more) overlap in variance among factors, hence, the need to do oblique rotation. Since our matrix shows values greater than this threshold, we further executed a promax rotation on the data to identify and name the measured variables that loaded on the retained components.

Appendix F shows the factor loading communalities for the 5 factors that were retained from the factor analysis and rotated using promax method to allow for ease of analysis and naming. The factors were named based on the majority of items that loaded for those factors. The full details of the measured variables based on the survey tool can be found in Appendix A. Measured variables that had loading coefficients less than 0.4 were suppressed during the factor analysis hence only variables with loading above 0.4 were returned. The results show that usefulness of EMR based on the survey item “Overall, I believe EMR will be useful for my job at my medical facility” had the highest factor loading. Meanwhile the lowest loading was for the item “EMR will facilitate patient consultations within my medical facility”.

### Regression analysis

In order to choose the type of regression analysis we could perform on the dataset; we needed to determine if the data was normally distributed or not. Since we had over 100 respondents (1,177 respondents), test of normality was performed on the data using the Kolmogorov-Smirnov test which is used for datasets greater than 100. The variables were transformed by calculating the means and the test for normality performed on the transformed data. The results showed that all variables were significant (*p* < 0.05) hence indicating that the data was not normally distributed. Since the data is not normally distributed, we carried out ordinal regression analysis instead of linear regression analysis.

The ordinal regression analysis model fitting information (Table 2) shows a significant improvement in fit of the final model over the null model [*χ*^2^(107) = 1,169.904, *p* < .001]. Meanwhile the goodness-of-fit test results show a Pearson chi-square test [*χ*^2^ (7,981) = 12,284.829, *p* < .001] and Deviance chi-square test [*χ*^2^ (7,981) = 1,957.612, *p* > .05]. The non-significant test result indicate that the model fits the data well (37). The Pseudo R-square value (Nagelkerke value) for this test (R^2^ = .674) indicates that 67.4% of the variance in user intention to adopt and use EMR can be explained by the independent variables considered in this study. The test of parallel lines was not significant (*p* > .05) hence indicating that we have not violated the test of proportional odds, hence the results obtained from the ordinal regression can be used to expound the impact of the independent variables on user intention to adopt and use EMR.

**Table 2:** Ordinal regression results.

Item	Measure	Value	DF	Sig
Model Fitting info	Chi-square *χ*^2^	1,169.904	107	.000
Goodness-of-fit	Pearson χ^2^	12,284.829	7,981	.000
	Deviance χ^2^	1,957.612	7,981	1.000
Test of parallel lines	General χ^2^	111.704	749	1.000
Pseudo R-squared	Nagelkerke	.674		

In order to confirm the results from the ordinal regression analysis before proceeding to interpret the results, we further carried out the ordinal regression using generalised linear model and ordinal logistic response. The results were consistent with the initial ordinal regression analysis but additionally provided a test for the proposed model constructs to indicate which variables were statistically significant in influencing user decision to adopt and use EMR. The model test results show that awareness, risk, relative advantage, usefulness and critical success factors were statistically significant as factors that impact adoption of EMR. Meanwhile, infrastructure, data security and ease of use were not statistically significant in influencing user adoption of EMR, see Table 3. We further performed a test for collinearity to ensure that the variables are not highly correlated. The tolerance values for all the independent variables were above the 0.10 threshold and the variance inflation factor (VIF) was less than the maximum permitted value of 10.0 (38). This therefore indicates that our data was not correlated and can be used to analyse the impact of the independent variables on our dependent variable (intent to adopt EMR).

**Table 3:** Model test.

Variable	Coefficient *β*	Exp (*β*)	Chi-Square χ^2^	Tolerance	VIF	Sig
Awareness	.422	1.525	13.911	.640	1.562	.000
Risk	−.516	.597	35.308	.719	1.391	.000
Infrastructure	.079	1.082	.897	.577	1.733	.344
Data security	−.134	.875	1.929	.547	1.828	.165
Relative advantage	.356	1.427	8.429	.492	2.032	.004
Ease of use	.110	1.116	.362	.372	2.688	.547
Usefulness	2.910	18.357	278.476	.411	2.432	.000
Critical success factors	.877	2.404	57.972	.582	1.718	.000

The Exp(β) values represent the odds ratios that reflect the multiplicative changes in the odds of being in a higher category on the dependent variable for every one unit increase in the dependent variable, holding all other independent variables constant. So, odds ratios greater than 1 will represent increasing probability of impacting the dependent variable while odds ratios of less than 1 will represent decreasing probability of impacting the dependent variable. The results therefore show that usefulness has the highest impact to increase adoption of EMR with an odds ratio of 18.357. This indicates that user propensity to adopt EMR increases by a factor of 18.357 for every one unit increase in user perception of its usefulness. This is followed by critical success factors with odds ratio of 2.404, awareness with odds ratio of 1.525 and relative advantage with odds ratio of 1.427. Even though infrastructure availability and ease of use have odds ratios above 1, they are not statistically significant. Meanwhile, risk and data security both have odds ratios below 1 with negative coefficients. This indicates that user perception of risk and safety of their data when using EMR decreases their propensity to adopt EMR.

### Mediation and moderation tests

In our proposed model, we posited that relative advantage will mediate user perception of usefulness of EMR. We also posited that age, gender and experience have moderating effects on user intention to adopt and use EMR. The results of the mediation and moderation analysis are shown in Appendix G and Appendix H respectively. The results show that the direct impact of both usefulness and relative advantage on adoption and usage of EMR are significant (*p* < .001). To calculate the mediation effect of relative advantage on usefulness of EMR, a Sobel test (39) was conducted to determine if this mediation was significant and the mediation coefficient was also computed (see row UFxRA≫AD in Appendix F) resulting a positive coefficient of 0.096. Hence, we can conclude that relative advantage has a positive significant mediating impact on usefulness of EMR.

The moderation test was done by calculating the interaction term between the moderating variable and the moderated variable and then using SPSS to run a simple linear regression on the variables (Appendix H). The moderation test revealed that age, gender and experience do not have direct significant impact on user decision to adopt and use EMR. However, gender has a significant impact (*p* < .05) on user perception on ease of use of EMR and hence influence it adoption. Also, experience has significant impact (*p* < .05) on user perception of risk when it comes to the adoption of EMR. Meanwhile, the moderation effects of age on usefulness, age on ease of use, gender on usefulness, gender on risk, experience on ease of use and experience on critical success factors were statistically not significant thereby rejecting the hypotheses that posited that these factors will have a moderating impact on user decision to adopt and use EMR.

### Hypotheses testing

The results from the model test using ordinal regression analysis, mediation test and moderation tests were used to test the hypotheses that were propounded in the conceptual design. Table 4 provides the results of the hypotheses tests and shows that eight of the proposed hypotheses were supported (*p* < 0.05). Usefulness of EMR (H1) had the highest coefficient thereby indicating that users are more inclined to adopt and use EMR due to its usefulness. This was followed by critical success factors (H8) which indicates that users will only adopt EMR if it can fulfil certain conditions that will ensure success of their operations. Surprisingly, ease of use (H2) was not deemed to be very crucial for the adoption and usage of EMR hence the hypothesis was not supported despite many prior studies showing that ease of use is relevant to user adoption and use of new technology. The mediation test shows that relative advantage does have a significant positive impact on the usefulness of EMR (*β* = 0.096, *p* = 0.000). The results also show that age did not have any moderating impact on usefulness or ease of use of EMR meanwhile gender significantly mediates the impact of ease of use of EMR and experience was also found to significantly mediate perception of risk in the adoption and use of EMR. It is also worth noting that risk (H5) has a significant negative impact on user decision to adopt and use EMR. This is consistent with prior adoption studies which have highlighted user reluctance to adopt new technology if there is a perception of risks to their operations or data.

**Table 4:** Hypotheses test results.

Hypothesis	Path	Path Coefficient	*p*-value	Hypothesis Status
H1	**UF → AD**	**2** **.** **910**	**.** **000**	**Supported**
H2	EU** → **AD	.110	.547	Not Supported
H3	**RA → AD**	**.** **356**	**.** **004**	**Supported**
H4	**AW → AD**	**.** **422**	**.** **000**	**Supported**
H5	**RS → AD**	**−0** **.** **516**	**.** **000**	**Supported**
H6	DS → AD	−0.134	.165	Not Supported
H7	IF → AD	.079	.344	Not Supported
H8	**CS → AD**	**.** **877**	**.** **000**	**Supported**
H9	**UFxRA → AD**	**.** **096**	**.** **000**	**Supported**
H9a	AGE_UF → AD	−0.016	.480	Not Supported
H9b	AGE_EU → AD	−0.010	.732	Not Supported
H9c	GEN_UF	−0.064	.171	Not Supported
H9d	**GEN_EU**	**−0** **.** **191**	**.** **001**	**Supported**
H9e	GEN_RS	.059	.194	Not Supported
H9f	EXP_EU	−0.018	.391	Not Supported
H9g	**EXP_RS**	**−0** **.** **050**	**.** **003**	**Supported**
H9h	EXP_CS	−0.007	.694	Not Supported

UF, Usefulness; AD, Adopt & Use EMR; EU, Ease of use; RA, Relative advantage; AW, Awareness; RS, Risk; DS, Data security; IF, Infrastructure; CS, Critical success factors; GEN, Gender; EXP, Years of experience.

Bold letters represent statistically significant values.

## Discussion

Poor health information system has been identified as a major challenge in the health-care system in many sub-Saharan African countries including Nigeria. Although, EMR is an important tool to improve access to patient information with attendant improved quality of care, EMR has not been widely implemented/adopted in sub-Saharan Africa especially Nigeria. Thus, this study sought to identify factors that affect the adoption of EMR in Nigeria.

The demographics of this study reveals some interesting findings about the Nigerian health sector. In this study, we deliberately set out to study a good mix of decision makers and system users. Majority of the respondents were female, between the 31–50 age group. Most of the medical facilities were in the public sector. While this is consistent with the age of the working class in Nigeria, the female preponderance presupposes that there are more females in the facilities. However, further analysis suggests they are not equitably distributed in the different cadres, being skewed towards the lower cadres as compared to administrative/managerial positions (see Appendix I). This is in keeping with studies that show that although women make up a large proportion of the health workforce, very few are in senior managerial/leadership positions (40). It was interesting to see that while most of the medical facilities depended on the administration to make decisions on new technology adoption, very few (6.7%) relied on their IT department for such decisions. The reasons may be due to the weak ICT systems currently in place as most of the facilities had ICT units manned by only one or two staff as opposed to having IT departments.

Although the study targeted administrators and those who were able to make decisions in the system, the researchers were surprised to see that only 28% of the respondents were medical doctors. This confirms the global shortage of physicians and the capital flight of physicians in Nigeria.

Our study results also show that the majority of respondents had intermediate level of computer literacy which can directly impact their willingness to adopt and use EMR systems. This is consistent with studies conducted on computer and internet use by doctors in a Nigerian teaching hospital which revealed that the overall proficiency of the respondents in computer-based competencies was below average with only 26.7% sufficiently familiar with computer tools to perform advanced tasks (18, 41). A study of three hospitals in Ethiopia also revealed that health professionals who were computer literate are more likely to adopt EMR than their counterparts who are not computer literate.

In our study, usefulness of new technology was an important facilitator to user willingness to adopt and use the technology. It had the highest impact to increase adoption of EMR with an odds ratio of 18.357, which indicates that user propensity to adopt EMR increases by a factor of 18.357 for every one unit increase in user perception of its usefulness. This is comparable with a study done in Taiwan among Nurses (42) and in another study done in Sri Lanka, where human factors such as lack of awareness on the benefits of EMR, lack of knowledge and experience on how to use EMR, negative perceptions and attitudes on EMR were vital barriers to successful implementation and adoption of EMR (43). Also, a study among Physicians in Iran showed that perceived usefulness has a direct and significant effect on physicians' attitudes toward EMRs' adoption (44). A systematic review by Ross et al. (9) showed that usefulness was an important factor in adoption of EMR. These however contrast with a study in Skopje, Macedonia among health professionals which showed that usefulness of a new technology was not an important factor in its adoption (45).

Perceived ease of use and awareness which are important factors for adoption in this study have been linked with training, knowing the benefits of EMR, access to information, improved knowledge and experience of EMR. Surprisingly, ease of use (H2) was not deemed to be very crucial for the adoption and usage of EMR hence the hypothesis was not supported despite many prior studies showing that ease of use is relevant to user adoption and use of new technology (43). A study on acceptance of health information technology among health professionals revealed that ease of use was an important factor on the adoption of EMR (45). In other studies, ease of use was a significant contributor to adoption of EMR (42, 46).

Critical success factors that guide management and stakeholders' decisions to adopt new technology was significant in this study and had an odds ratio of 2.404 which indicates that user propensity to adopt EMR increases by a factor of 2.404 for every one unit increase in user perception of its critical success factor. Management and stakeholder engagement and support increases adoption and implementation of EMR through fostering a sense of ownership, building confidence, acceptance, enjoyment and self-pride (9). Organizational leadership had been shown to play a great role in providing resources to adopt and use EMRs. Results from a study on Jordan hospitals revealed a positive relationship between management support and adoption of new technology (47). Pantuvo et al. (48) revealed that the critical success factors for adoption and implementation of EMR in Nigeria were enforceable legislation.

Awareness with odds ratio of 1.525 was significant and indicates that user propensity to adopt EMR increases by a factor of 1.525 for every one unit increase in user awareness. Previous study had linked awareness to having good attitude towards EMR. A study of three hospitals in Ethiopia showed that health professionals who had good attitude towards EMR were 1.56 times more likely to readily adopt EMR compared to those health professionals who had poor attitude (3). Access to information and knowledge increases the adoption of EMR (41). This may be due to the fact that health professionals who have information on EMR or witnessed EMR being used before with the visibility of its benefit may have the tendency to accept the advantage of technology and likely to be ready for EMR adoption. Relative advantage, which is the way new technology will perform better than the one being replaced, will also influence decisions to adopt the technology. In this study, relative advantage had an odds ratio of 1.427 which indicates that user propensity to adopt EMR increases by a factor of 1.427 for every one unit increase in user perception of its relative advantage. This is similar to the study by Scott et al. (49) on uptake of the Canadian Heart Health Kit which found that relative advantage significantly influences physicians' intention to use the new innovation. The mediation test shows that relative advantage also has a significant positive impact on the usefulness of EMR.

Previous authors had described available resources to include the availability of suitable infrastructure important for implementation of EMR successfully. Infrastructure features included electricity supply, available bandwidth, access to reliable internet connectivity, access to computers, electrical power and access to phone lines and mobile phones (9). In Nigeria, lack of constant supply of electricity (48) as well as poor internet services are barriers to successful adoption and implementation of a nationwide EMR (41). Infrastructural availability is a major inhibitory factor to the adoption of EMR in Nigeria as documented by other studies but was found not to be statistically significant in our study. Many hospitals in Nigeria depend mainly on alternative power supply commonly called “generator” for their operations, EMR may not be used consistently because of the constant power outage. Also, internet connectivity is very low in Nigeria. A study conducted on the use of health information and communication technologies by health workers in seven state hospitals and a private hospital in the South Western Zone, Ogun State, Nigeria, reported that only one of the hospitals examined was connected to the internet, and none of them had a website (50). Previous study showed that health professionals who believed that there was good technical Infrastructure for EMR system were 1.78 times more likely to adopt EMR system (3).

In this study risk and safety of data were negatively correlated with adoption of EMR as they both had negative coefficients. This indicates that user perception of risk and safety of their data when using EMR decreases their propensity to adopt EMR. This is comparable with a systematic review on factors that influence the implementation of e-health, where concerns over patients’ privacy, security threats and perceived threats over patients and health professionals' relationship were seen as barriers to adoption of EMR (9). A study on factors affecting the adoption of EMR by nurses in Tamil Nadu, India, showed that risk was a barrier to adoption of EMR (46). In this study, the moderation test revealed that age, gender and experience do not have direct significant impact on user decision to adopt and use EMR. This is in contrast to a study done in Ethiopia that revealed that health professionals aged 30–34 years were 52% less likely to be ready for EMR system than younger health professionals (3) and another systematic review that showed that younger primary care physicians were more inclined to use EMR than older physician (13). This maybe so because younger respondent had multiple access to internet than older respondent (51). More so, younger people naturally tend to have more motive, interest, and readiness to accept new technology developments than older people (3).

The results of our study also showed that age did not have any moderating impact on usefulness or ease of use of EMR. Biruk et al. (3) found out that male health professionals were 1.87 times more likely to be ready to adopt EMR system than female health professionals. However, in our study, gender had a significant impact (*p* < .05) on user perception on ease of use of EMR and hence influence it adoption. This may be so because several studies have shown that males are more proficient in computer use than females (41, 51). Also, experience has significant impact (*p* < .05) on user perception of risk when it comes to the adoption of EMR.

## Conclusion

This study set out to explore the factors that influence the decision of healthcare professionals to adopt and use an EMR system in developing countries. A conceptual adoption model was developed based on an extended TAM model which included constructs of awareness, risks, data security, infrastructure and relative advantage to investigate the propensity of stakeholders in Nigeria to adopt and use electronic medical records. The results of our model testing shows that usefulness has the highest impact on adoption of EMR. This is followed by critical success factor, awareness and relative advantage. However, though infrastructure availability and ease of use were also important in the model, they were not statistically significant. Meanwhile, user perception of risk and safety of their data when using EMR decreases user propensity to adopt EMR.

Our results suggest that the Nigerian health care delivery system is ready to adopt EMR. However, several challenges remain for the successful adoption and implementation of EMR in the Niger Delta region and by implication similar contexts in developing countries. In this study, infrastructural availability being lack of or poor internet connectivity and lack of constant supply of electricity were not seen as barriers to successful adoption and implementation of an EMR system. While this may be due to dependence by many hospitals in Nigeria on alternative power supply for their operations, however, this remains a huge challenge, especially in peripheral facilities as EMR may not be used consistently because of the constant power outage. Lack of functional websites is also a challenge for majority of the hospitals.

### Policy recommendations

We therefore make the following policy recommendations:

#### Government

The key role of the government in health would be improved formulation of policies on EMR adoption with regulatory bodies to oversee its implementation. In Nigeria, EMR is a new technology in the nascent stages of adoption in several states. Regulation is also rudimentary. The government thus should be actively involved, designating appropriate agencies to partner with the federal ministry of health to develop regulatory policies and guidelines. More so, there should be increased funding to health and functional health records department in all health facilities. The government, management and stakeholders should ensure improvement of infrastructures for the adoption, implementation and sustainability of EMR. Stable electricity to health facilities should be prioritized. Government should fast-track the development of an e-health infrastructure, such as internet backbone and satellite technology, as well as the spread of high-speed broadband data services. Develop legislative and implementation frameworks for public-private partnerships in the health sector in order to attract private sector investment and ensure the sustainability of EMR projects. Regulation of data safety, protection and Risk management should be addressed to encourage stakeholder buy-in for adoption of EMR.

#### Healthcare facility management

The management should also prioritize and invest in EMR implementation and maintenance of innovative EMR systems that will lead to more efficient service delivery at their facilities. The investment will cover training, infrastructure installation and maintenance. Management should set up an effective system that will deal with feedbacks promptly.

In preparation for the adoption of digital interventions especially EMRs in LMICs, hospital management need to be ready to commit the resources and investments to make them successful. Topmost on the priority list would be electrical power supply and deploying adequately trained staff to run functional ICT departments. This would be key to the smooth functioning of any EMR system.

Critical success factor such as early involvement of stakeholders to build up the requirements of end users and reduce resistance to change is highly recommended. The perceived benefits of EMR should be identified and communicated to stakeholders as much as possible. The hospital management should be involved in enforcing policies on EMR, training and motivating the workforce and have sustainable funding for EMR. Training and retraining of medical personnel should also be encouraged through in-school training on medical informatics, in-service training on ICT and continuing medical education.

To ensure effective implementation, Training of all personnel on management of the system should also be encouraged, besides in-service training on ICT medical informatics. There is also need to integrate in-school training on medical informatics in medical and allied medical courses as all members of the health team would be end users of any EMR system.

EMR system vendors should demonstrate its usefulness and ease of use to the healthcare workers beyond reasonable doubt. Safety of data should be sacrosanct. This should guide their decision on which type to EMR program to buy into. This process should be rigorously and painstakingly done because one breach is enough to lead to system collapse, loss of confidence and numerous litigations. The administrator should have a mechanism at inception of EMR that will work at identifying, categorizing and assessing cyber risks. Mitigation of risks should be prompt and demonstrable so as to engender confidence in the system.

#### Individual

At individual level, our results showed that critical success factor showed a positive impact on ease of adoption. This suggests that both the end users and administrators should understand and promote EMR systems, with adequate reference to successful systems to motivate their staff to adopt and use it. Improving awareness will encourage staff to invest their time and finances to educate and upgrade themselves in preparation for adoption of IT innovations which is critical to improved patient care and service delivery.

#### Research implications

We recommend further research on readiness to adopt other digital innovations for the health system including medical decision-making systems and telemedicine.

### Limitations and future research

This study was cross-sectional quantitative research focused only on the Niger Delta region of Nigeria. Due to budgetary and time constraints, it could not be extended to the western and northern states. However, we are of the opinion that the adoption challenges faced in the states selected for this study will be very similar to those faced across the whole country. We will therefore recommend further research with a national coverage. Our study also focused just on the adoption of EMR but there are many other medical decision support systems in use today. Hence, we also encourage future research to focus on adoption of medical decision support systems and telemedicine in similar contexts.

## Data Availability

The raw data supporting the conclusions of this article will be made available by the authors, without undue reservation.

## Appendix A Experimental variables

**Table:**

I have known about EMR for some time now	EMRAW1
I know of the possibilities and flexibilities offered by using EMR	EMRAW2
I have used EMR before	EMRAW3
I am currently using EMR within my medical facility	EMRAW4
EMR will lead to time saving managing patient records	EMRAW5
EMR will facilitate patient consultations within my medical facility	EMRAW6
I feel that EMR are safe and secure to use	EMRRS7
I believe that there are no risks when using EMR	EMRRS8
I believe that my medical facility has the necessary infrastructure needed to adopt EMR	EMRIF9
I believe that patient data stored on EMR is safe and secure	EMRDS10
I believe that using EMR will give my medical facility an edge over others not using it	EMRRA11
I believe interaction with EMR will be clear and understandable	EMREU12
I believe it will be easy for EMR to do what I want from it	EMREU13
I believe that learning to use EMR will be easy for me	EMREU14
Overall, I believe that EMR will be easy to use	EMREU15
Using EMR will improve the quality of the work I do at my medical facility	EMRUF16
Using EMR will give me greater control over my patients’ records	EMRUF17
Using EMR will enable me complete tasks quickly	EMRUF18
Using EMR will increase productivity for staff at my medical facility	EMRUF19
Overall, I believe EMR will be useful for my job at my medical facility	EMRUF20
I will recommend adoption and usage of EMR at my medical facility	EMRAD21
I intend to use EMR system when implemented at my medical facility	EMRAD22
My medical facility has the appropriate change management process to facilitate the adoption of EMR system	EMRCS23
My medical facility's business culture will not interfere with the adoption of the EMR software	EMRCS24
In my opinion, the top management in my medical facility support the adoption of EMR software	EMRCS25
There is an effective communication structure in my medical facility to encourage adoption of EMR software	EMRCS26
My medical facility business vision and missions supports the adoption of EMR software	EMRCS27
The EMR software is appropriate for our business functions	EMRCS28
My medical facility has the resources required to support and maintain EMR software	EMRCS29
In my opinion, the initial costs will not inhibit the adoption of EMR	EMRCS30
In my opinion, the operating costs will not inhibit the adoption and use of EMR	EMRCS31

## Appendix B KMO and Bartlett's test

**Table:**

Kaiser-Meyer-Olkin measure of sampling adequacy		.884
Bartlett's Test of Sphericity	Approx. Chi-square	5,043.015
	DF	465
	Sig.	.000

## Appendix C Total variance explained

**Table:**

Component	Initial Eigenvalues			Rotation Sums of Squared Loadings		
	Total	% of Variance	Cumulative %	Total	% of Variance	Cumulative %
1	12.329	39.772	39.772	7.233	23.332	23.332
2	3.064	9.883	49.655	5.911	19.069	42.401
3	1.937	6.248	55.902	2.540	8.193	50.595
4	1.514	4.884	60.786	2.513	8.108	58.703
5	1.266	4.085	64.870	1.912	6.168	64.870
6	.956	3.083	67.953			
7	.892	2.878	70.832			
8	.757	2.440	73.272			
9	.699	2.254	75.527			
–	–	–	–			
–	–	–	–			
29	.204	.659	98.935			
30	.193	.621	99.556			
31	.138	.444	100.000			

Extraction Method: Principal Component Analysis.

## Appendix E Component correlation matrix

**Table:**

Component	1	2	3	4	5
1	1.000	.549	.541	.453	.082
2	.549	1.000	.395	.328	.242
3	.541	.395	1.000	.359	−.085
4	.453	.328	.359	1.000	.202
5	.082	.242	−.085	.202	1.000

Extraction Method: Principal Component Analysis. Rotation Method: Promax with Kaiser Normalization.

## Appendix F Factor loading communalities for five-factor principal component analysis with promax rotation EMR adoption items

**Table:**

Label	Usefulness of EMR	EMR Critical success	Awareness of EMR	Risks, Data security & Infrastructure	Usage Experience
Items	Factor 1	Factor 2	Factor 3	Factor 4	Factor 5
EMRUF20	.893				
EMRUF19	.860				
EMREU14	.850				
EMRUF18	.817				
EMREU15	.805				
EMRUF16	.792				
EMRUF17	.787				
EMREU13	.701				
EMREU12	.635				
EMRAD22	.622				
EMRAD21	.619				
EMRRA11	.536				
EMRCS26		.849			
EMRCS27		.815			
EMRCS30		.803			
EMRCS24		.788			
EMRCS29		.775			
EMRCS31		.772			
EMRCS25		.765			
EMRCS23		.744			
EMRCS28		.690			
EMRAW1			.893		
EMRAW2			.843		
EMRAW5			.551		
EMRAW6			.413		
EMRRS8				.862	
EMRRS7				.675	
EMRDS10				.624	
EMRIF9				.453	
EMRAW3					.877
EMRAW4					.798

Extraction Method: Principal Component Analysis. Rotation Method: Promax with Kaiser Normalization. a. Rotation converged in 6 iterations.

## Appendix G Mediation effects

**Table:**

Model	B	Std. Error	t	Sig.
UF≫RA	.762	.028	27.345	.000
RA≫AD	.126	.024	5.358	.000
UF≫AD	.737	.029	25.596	.000
UFxRA≫AD	.096	.018	5.155	.000

## Appendix H Moderation effects

**Table:**

Model	B	Sig.	R^2^	R^2^ Change
AGE≫AD	.018	.215	.533	
AGE_UF≫AD	−0.016	.480	.533	.000
AGE_EU≫AD	−0.010	.732	.334	.000
GEN	−0.045	.103	.534	
GEN_UF	−0.064	.171	.534	.001
**GEN_EU**	**−0.191**	**.** **001**	**.** **334**	**.** **006**
GEN_RS	.059	.194	.020	.001
EXP	−0.004	.737	.334	
EXP_EU	−0.018	.391	.334	.000
**EXP_RS**	**−0.050**	**.** **003**	**.** **019**	**.** **007**
EXP_CS	−0.007	.694	.299	.000

## Appendix I Gender vs. Job title and rank classification

**Table:**

	RANK									
GENDER	OFF	REG	SR REG	CON	SR CON	CF CON	MED OFF	SR MED OFF	PRIN MED OFF	TOT
MALE	10	21	33	41	7	12	76	14	4	218
FEMALE	3	15	13	16	7	2	46	7	2	111
	**JOB TITLE**									
**GENDER**	**ADM**	DOC	**NUR**	**PHARM**	**LAB SCI**	**OTH**	**TOTAL**			
MALE	41	218	60	60	69	14	462			
FEMALE	42	111	375	59	96	32	715			
